# Attention Modulation to Linguistic Speech Units

**DOI:** 10.1162/nol.a.14

**Published:** 2025-09-05

**Authors:** Manuela Jaeger, Elana Zion Golumbic, Martin G. Bleichner

**Affiliations:** Neurophysiology of Everyday Life Group, Department of Psychology, Carl von Ossietzky Universität Oldenburg, Oldenburg, Germany; Gonda Multidisciplinary Brain Research Center, Bar-Ilan University, Ramat Gan, Israel; Research Center for Neurosensory Science, Carl von Ossietzky Universität Oldenburg, Oldenburg, Germany

**Keywords:** electroencephalography (EEG), lexical processing, neural speech tracking, selective auditory attention, speech processing, temporal response function (TRF)

## Abstract

This study investigates how selective auditory attention influences the lexical speech segmentation process to phonemes and words in a two competing speaker scenario. Using electroencephalography recordings from 20 participants, we applied temporal response function analysis to distinguish attention-driven neural activity to phoneme and word onsets for the attended and ignored speech stream separately. Our results reveal distinct attention effects for phoneme and word onsets. Phoneme onsets elicited significant selective attention effects at an early (18–94 ms, P1), middle (186–252 ms, P2), and late (302–382 ms, N2) time window. In contrast, word onsets showed attention effects only at a middle (192–280 ms, P2) and late (348–386 ms, N2) time window, occurring slightly later than phoneme-related effects. Prediction accuracy analyses demonstrated stronger model performance for the attended speech stream across all models, with notable improvements in prediction accuracy from a word model to a phoneme model to a combined word and phoneme model. These findings are in accordance with both hierarchical and parallel processing frameworks, where selective attention enhances lexical segmentation for attended speech, improving prediction accuracy. Early attention effects observed for phoneme onsets underscore their role in low-level speech processing, while late attention effects for word onsets may reflect higher level processing. This study highlights the importance of selective attention in neural speech tracking and provides insights into auditory processing mechanisms underlying speech comprehension in complex acoustic environments.

## INTRODUCTION

Typical human interactions take place in acoustically complex environments. Despite these adversities, the human auditory system copes remarkably well with these challenges, enabling us to follow a speaker of interest among concurrent other speakers (Cherry, 1953). In such demanding listening situations, speech understanding relies on the listener’s ability to segregate an auditory scene into separate auditory objects and on the ability to focus on a relevant sound stream while suppressing irrelevant sound information. Attention paid to a specific sound object facilitates auditory processing and resolves competition between multiple sources (Bizley & Cohen, 2013; Shinn-Cunningham & Best, 2008). Robust modulations of event-related potentials suggest that selective attention may be realized as a top-down sensory gain-control mechanism that enhances the responses to the attended auditory stimulus and/or downregulates the processing of the ignored stimulus (Choi et al., 2013; Hillyard et al., 1973; Jaeger et al., 2018; Woldorff et al., 1993). Given the limited capacity for linguistic processing of concurrent speech streams, this gain-control mechanism likely plays a crucial role in gating relevant sound information from early sensory to higher order brain regions (Bronkhorst, 2015). However, speech processing is based on a complex network of brain regions specialized for analysing different hierarchical levels of speech including phonological, lexical, and semantic processes. While existing hierarchical (Friederici, 2011; Hickok & Poeppel, 2007; Scott & Johnsrude, 2003) and parallel (Giraud & Poeppel, 2012; Pulvermüller & Fadiga, 2010) models of speech processing acknowledge this complexity, they do not make explicit predictions about the levels at which selective auditory attention modulates cortical speech processing. Therefore, whether the top-down sensory gain-control mechanism primarily operates at an early or late stage involving higher level linguistic processes remains not fully understood. Neural speech tracking offers a unique window into these dynamics, allowing us to examine how selective attention influences cortical speech processing at different hierarchical levels of speech.

Neural speech tracking refers to the phenomenon whereby the electrical brain activity time locks to certain aspects of the speech signal (for a review, see Brodbeck & Simon, 2020). One prominent speech feature is the speech envelope, which follows the slow amplitude fluctuations in a continuous speech signal over time. It has been found that the speech envelope of single speech stream is represented in ongoing auditory cortex activity, enabling the investigation of speech processing to continuous speech streams (Aiken & Picton, 2008; Kubanek et al., 2013; Luo & Poeppel, 2007; Nourski et al., 2009). However, real-world auditory environments are rarely limited to a single speech stream. Instead, they often involve multiple competing speech sources, as in the “competing speaker paradigm” described by Broadbent (1952). In this scenario, listeners must selectively focus on one speaker while ignoring the other, a task that poses significant challenges for auditory perception and cognitive processing as both speech streams are intelligible and semantically correct. Such situations require the brain to separate overlapping acoustic speech signals, maintain attention to relevant information, and suppress irrelevant information. Studying neural speech envelope tracking in a competing speaker paradigm allows researchers to investigate how the brain manages selective auditory attention in a complex auditory environment. When one speaker is attended while the other is ignored, differences in cortical tracking of the speech envelopes provide valuable insights into how selective attention modulates neural speech processing. Specifically, selective attention to one of two speech streams results in a stronger cortical phase-locking to the attended compared to the ignored speech envelope (Ding & Simon, 2012; Fiedler et al., 2019; Horton et al., 2013; Kerlin et al., 2010; Kong et al., 2014; Mesgarani & Chang, 2012; Zion Golumbic et al., 2013).

Linguistic research shows that multiple distinct representation levels link speech sounds to comprehension, a process that relies on successful speech segmentation (Friederici, 2011; Scott & Johnsrude, 2003). Speech segmentation refers to how the human brain identifies boundaries between speech units, such as phonemes, and words. Phonemes, the smallest linguistic units, are the building blocks of words, and their segmentation is crucial for successful speech comprehension. Even though recent models of speech processing (Friederici, 2011; Giraud & Poeppel, 2012; Hickok & Poeppel, 2007; Pulvermüller & Fadiga, 2010; Scott & Johnsrude, 2003) differ in their organization, they all emphasize that different, yet interconnected, neural circuits are involved in processing phonemes and words. Phonemes are processed at early sensory levels within the auditory pathway, while words are processed through a more advanced hierarchy that involves higher level syntactic and semantic linguistic integration. When exploring the link between neural activity and speech processing, the speech envelope has proven to be a valuable feature for tracking how the brain processes continuous speech (Brodbeck & Simon, 2020). The speech envelope reflects slow, rhythmic intensity modulations that capture prosody and speech rhythm (Myers et al., 2019). Importantly, this approach can be extended beyond the acoustic envelope properties of speech by combining it with other key features, such as spectrotemporal cues (Daube et al., 2019; Di Liberto et al., 2015), phonemes (Brodbeck et al., 2018; Di Liberto et al., 2015; Gillis et al., 2021), words (Brodbeck et al., 2018; Gillis et al., 2021; Weissbart et al., 2020), and higher level linguistic representations (Agmon et al., 2023; Broderick et al., 2018; Donhauser & Baillet, 2020; Koskinen et al., 2020). These features are independent from the speech envelope information and can therefore capture more complex aspects of speech processing such as the information content of words, or words at syntactic boundaries (Agmon et al., 2023). This combined approach has been used, for example, to study speech intelligibility (Verschueren et al., 2022) and language comprehension (Broderick et al., 2022; Mesik et al., 2021), providing insights into how these speech features are processed by the brain to enable successful speech comprehension. Furthermore, research has shown that selective auditory attention modulates neural responses to low and higher level linguistic features differently (Brodbeck et al., 2018; Broderick et al., 2018; Mesik et al., 2021), suggesting that the brain dynamically prioritizes different information streams based on the demands of the listening context. This approach is particularly useful in paradigms involving competing speakers, as it allows for the investigation of how the low- and high-level features of both the attended and ignored speech stream are processed.

In an attempt to replicate and extend recent results, we analysed an existing dataset to understand how phoneme and word level information are processed in the brain. Specifically, we investigated electroencephalography (EEG) responses to continuous speech streams to understand how selective auditory attention influences the lexical speech segmentation process in a two competing speaker scenario. We examined the neural tracking of speech by using a forward modeling approach that results in a temporal response function (TRF) and a prediction accuracy for each EEG channel. While a TRF represents a linear approximation of the brains impulse response to the speech stimulus, the prediction accuracy is considered a measure of neural tracking. The higher the prediction accuracy, the better the stimulus is represented in the ongoing brain activity. To disentangle selective attention effects, we examined the TRF to phoneme and word onsets in the attended and ignored speech stream separately. We used a more parsimonious approach and differentiated between low and higher level features, where we take phonemes as a low-level feature and words as a high-level feature. To control for the basic acoustic properties of the speech, we included the speech envelope as an additional regressor in the forward models (Gillis et al., 2021). The unique contribution of lexical speech segmentation was quantified by analysing the increase in prediction accuracy from a word-to-phoneme onset regressor model and from when the word onset regressor is added to a model including the phoneme onset regressor. We hypothesized selective auditory attention effects to be reflected in the TRFs to phoneme and word onsets: (1) Based on models of hierarchical speech processing we expected early selective attention effects in the TRF response to phoneme onsets and late selective attention effects in the TRF response to word onsets. (2) We expected effects of lexical speech segmentation to be reflected as a stronger increase in prediction accuracy in the attended compared to the ignored speech stream.

## MATERIALS AND METHODS

### Participants

The current study analysed a previously recorded cap EEG dataset originally used in Jaeger et al. (2020) to investigate the feasibility of an online processing pipeline for decoding auditory selective attention based on speech envelope tracking. Twenty-one native German speaking participants between the ages of 19 and 30 (mean age = 22.3 yr; *SD* 2.7; 16 female) took part in the study. All participants reported no present neurological or psychiatric conditions. Audiometric thresholds of 20 dB HL or better in both ears were confirmed by pure tone audiometry at octave frequencies from 250 Hz to 8 kHz. The original study was approved by the local ethics committee (University of Oldenburg, Germany, Drs.Nr.27/2018) and conforms with the World Medical Association Declaration of Helsinki. All participants signed written informed consent prior to the experiment and received monetary reimbursement afterward. One individual had to be excluded from the analysis due to technical problems (data loss) during the experiment, leaving a sample size of *N* = 20 for the EEG analysis.

### Experimental Procedure

To investigate selective auditory attention effects in a demanding listening situation, we implemented a paradigm with two competing speakers similar to previously reported studies (Mirkovic et al., 2015; O’Sullivan et al., 2015). Participants were instructed to attend to one of two simultaneously presented speech streams throughout the entire experiment (approximately 60 min). One speech stream was presented from the right and the other from the left side to achieve a natural listening situation in which participants were able to use additional spatial cues to direct selective auditory attention. The to-be-attended speech stream and its side of presentation was not changed during the experimental session but was randomized across participants. The stimulus presentation consisted of six blocks lasting 10 minutes each and separated by short breaks. Before each stimulus presentation block, an arrow, presented on a screen, pointed in the direction of the to-be-attended speech stream to remind participants about the attended story and its side of presentation. In the stimulus presentation blocks participants were instructed to keep their eyes open and to focus their gaze on a white fixation cross on a light grey background. During short breaks participants were asked to fill out a multiple-choice questionnaire related to the content of each speech stream in the previous block. Participants were instructed to answer as many questions as possible but were discouraged from guessing the answers to any question by choosing to leave a question unanswered if they did not know the answer. Even the questionnaire contained questions related to both speech streams, participants were further encouraged to continue attending only to the indicated speech stream and to ignore the other one.

The two speech streams consisted of fairy tales narrated in German by two different professional male speakers. For each speech stream silent gaps longer than 500 ms were reduced to this length. The amplitude of both speech streams was adjusted to achieve equal loudness. A detailed description of the speech material and loudness adjustment is available in Mirkovic et al. (2016). Both speech streams were sampled at a rate of 48 kHz and presented to the participant using Psychophysics Toolbox for MATLAB (Brainard, 1997), a HDSP 9632 sound card (RME, Haimhausen, Germany), a ADI 8 DS MK III DA converter (RME, Haimhausen, Germany), PA5 attenuator (Tucker-Davis Technologies, Alachua, US), a C245BEE amplifier (NAD, Pickering, Canada), and two Sirocco S30 loudspeakers (Cambridge Audio, London, UK). The loudspeakers were located in front of the participant 45° to the right and to the left at ear height. The distance between loudspeaker and ear was 1.1 m. Simultaneous presentation of the two sound streams via loudspeakers resulted in a comfortable sound pressure level of 70 dB SPL, measured at the place of the participant’s head.

During the experiment, participants were comfortably seated in a sound-attenuated and dimly lit booth. EEG data were collected simultaneously from two different electrode layouts, a high-density EEG cap and two cEEGrids (Debener et al., 2015) placed around each ear of the participant. The cEEGrid data are not included in this analysis but have been presented elsewhere (Holtze et al., 2022). The high-density EEG cap consisted of 94 Ag/AgCl electrodes arranged in a customized, infracerebral electrode cap with an equidistant electrode layout (Easycap, Herrsching, Germany). Two additional electrodes were placed below the eyes to record electrooculograms. BrainAmp amplifiers (Brainproducts GmbH, Gilching, Germany) recorded all channels against a nose-tip reference with a sampling rate of 500 Hz and band-pass filtered the data from 0.0159 to 250 Hz. Electrode impedances were kept below 20 kΩ.

The experimental setup consisted of two personal computers connected with ethernet cable to a switch and building a small network. A presentation computer was responsible for auditory stimulus presentation, sound onset marker delivery, and presentation of visual instructions on a screen located in the booth. High density EEG cap and cEEGrids EEG signals as well as sound presentation onset markers were streamed into the network and integrated using the Lab Recorder software from the Lab Streaming Layer (LSL; Swartz Center for Computational Neuroscience & Kothe, 2015) package running on the recording computer. LSL enables the collection of time synchronized time series from different recording modalities.

### EEG Preprocessing

All analysis steps were performed in MATLAB (MathWorks, 2020), using customized scripts and the EEGLAB toolbox (Version 13.6.5b; Delorme & Makeig, 2004). EEG raw data preprocessing included the following steps. EEG channels, affected by the cEEGrid placement around the ears and EEG channels in the neck with larger distance to the skin were removed and spherically interpolated. Further, EEG data were re-referenced to common average, low-pass filtered at 40 Hz (FIR filter, filter order: 100, window type: Hann) and high-pass filtered at 1 Hz (FIR filter, filter order: 500, window type: Hann) to remove drifts from the data. The preprocessed EEG data were submitted to a processing pipeline performing EEG artifact attenuation using Artifact Subspace Reconstruction (ASR) as introduced by Mullen et al. (2013) and available as EEGLAB plugin clean rawdata (Version 0.32). ASR is based on a sliding-window principal component analysis, and it attenuates high-variance signal components in the EEG data (e.g., eye blinks, eye movements, and motion artifacts) relative to some artifact-free calibration data reasonably well (Blum et al., 2019). To derive the required artifact-free calibration data the first block of the EEG data recording was used. Time windows containing abnormally high-power artifacts were automatically removed from the preprocessed EEG data by running the clean window function. The function is included in the clean raw data plugin and was called based on default parameters except of the MaxBadChannels parameter: Aiming for a very clean output we used a value of 0.075. The obtained artifact-free calibration data were submitted to the ASR calibration method (function asr calibrate) to derive a state structure containing the statistical properties of the calibration data. This state structure was submitted together with the original preprocessed EEG data of all six sound presentation blocks to the ASR processing method (function asr process). During the processing step the ASR method detects artifacts based on their deviant statistical properties and linearly reconstructs the EEG data from the retained signal subspace based on the statistical properties of the calibration data. After artifact attenuation, the preprocessed EEG data were low-pass filtered at 15 Hz (FIR filter, filter order: 100, window type: Hann) and segmented into six trials, each 10 min in length, corresponding to the original sound presentation blocks. Before submitting the neural data to the speech tracking analysis, the multichannel EEG data were normalized (z-scored) together to preserve the relative power across channels. This normalization step aims for a more consistent tuning of model parameters across datasets (Crosse et al., 2021).

### Data Analysis: Speech Material

#### Speech annotation and derived regressors

**Figure 1.**
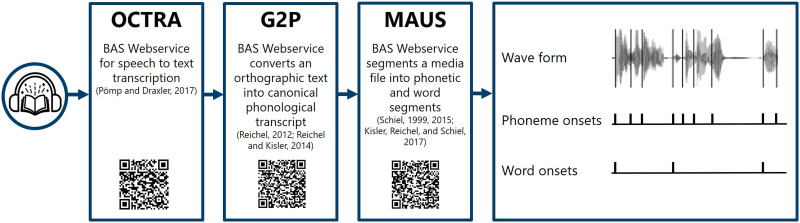
Speech annotation pipeline. The Munich Automatic Segmentation Web service (MAUS) was used to break down the continuous speech signal into discrete word and phoneme onsets. BAS = Bavarian Archive for Speech Signals.

Both audiobooks were submitted to a speech annotation pipeline to extract representations of speech segmentation, that is, time-aligned sequences of phonemes and words. The speech annotation pipeline consists of a set of different tools that are used one after the other (see Figure 1). OCTRA (Pömp & Draxler, 2017), G2P (Reichel, 2012; Reichel & Kisler, 2014), and Munich Automatic Segmentation (MAUS; Kisler et al., 2017; Schiel, 1999, 2015) are Web applications that are provided by the Bavarian Archive for Speech Signals (BAS) CLARIN centre in Munich and were developed for the multilingual processing of spoken language. While OCTRA was used separately, the tools G2P and MAUS were combined in one processing pipeline that performs the different processing steps in sequence automatically.

Speech-to-text transcription was done by using OCTRA (Pömp & Draxler, 2017). We used Google’s automatic speech recognition to process a selected audio sequence, and the resulting transcript had to be corrected manually to ensure that the text is an exact representation of the spoken narrative.

Grapheme-to-phoneme conversion was carried out using G2P, a Web application for converting orthographic text into a canonical phonological transcript corresponding to a standard pronunciation (Reichel, 2012; Reichel & Kisler, 2014). G2P reads the continuous speech-to-text transcript and estimates the most likely phoneme sequence that a standard speaker is expected to articulate in German.

Finally, we used MAUS, which enables a detailed analysis of linguistic and phonetic structures within audio data (Kisler et al., 2017; Schiel, 1999, 2015). MAUS’s forced alignment capabilities were used in breaking down the continuous speech signal into identifiable, discrete word and phoneme segments, providing us with start and end times for each segment. MAUS uses the canonical phonological transcript derived from the G2P annotation step and estimates the most likely phonetic segmentation and labeling in the speech signal. To increase temporal resolution, the output frame rate of the MAUS algorithm was set to 1 ms. To ensure that the segmentation and labeling worked out well, the output text file was manually checked in PRAAT (Boersma, 2001) for forced alignment errors. Forced alignment errors refer to cases where the segment edges or segment labels did not match the speech sound and may occur due to nonspeech noise like breathing. To improve the segmentation performance of the MAUS algorithm, we manually segmented the speech recording into separate intervals (speech chunks) in PRAAT. MAUS used this information to automatically split the speech sound according to the chunk information, segmented and labelled the speech in each chunk separately, and finally aggregated the output in a single output file. This process of manual correction was performed twice to reduce the amount of alignment errors. After submitting both audiobooks to the speech annotation pipeline, the resulting representations were one-dimensional arrays with impulses on the onsets of, respectively, phonemes and words.

To control for the basic acoustic properties of the speech, temporal speech envelopes of both audiobooks were extracted in MATLAB (R2020a; MathWorks, 2020) by computing the absolute values of the Hilbert transform. The transformed signals were low-pass filtered at 8 Hz (filter type: Butterworth, filter order: 4) using a zerophase filter based on processing the input data both in the forward and reverse directions. The resulting speech envelopes were downsampled to 500 Hz to fit the EEG sampling rate.

#### Stimulus characteristics/statistics

**Figure 2.**
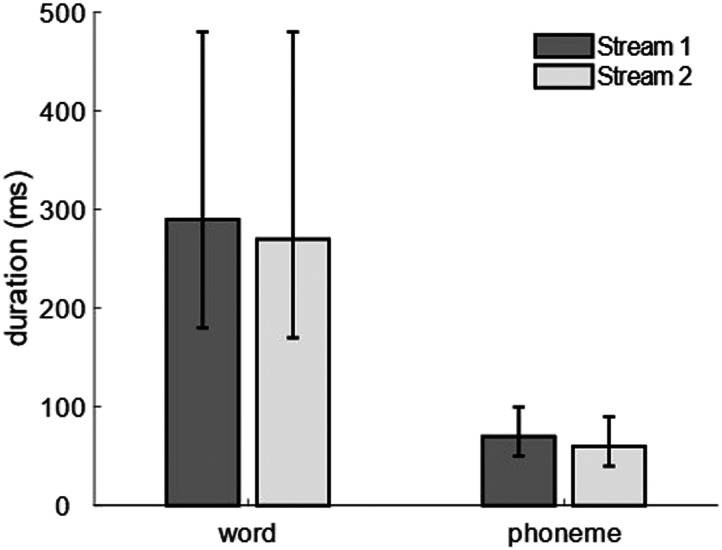
Median word and phoneme duration for speech stream 1 and speech stream 2 in milliseconds across the six presentation blocks. Whiskers represent the 25th and 75th quantile.

Stimulus characteristics, specifically median word and phoneme duration, were derived based on the one-dimensional arrays with impulses on the onsets of phonemes and words. Figure 2 represents in a bar plot the median word and phoneme duration in milliseconds separately for speech stream 1 and speech stream 2. For speech stream 1 the median word duration was 290 ms and the median phoneme duration 70 ms. In the speech stream 2 the median word duration was 270 ms and the median phoneme duration 60 ms.

### Data Analysis: EEG

#### Determination of neural speech tracking using mTRF toolbox

We used the mTRF toolbox (Crosse et al., 2016) to examine the neural tracking of speech by applying a linear forward modeling approach based on regularized linear regression that results in a TRF. The TRF describes the brain’s linear transformation of a stimulus feature to the continuous neural response over different time lags. The time lags ranged from 0 to 500 ms, a time range suitable to track higher order speech processing (Brodbeck et al., 2018). To reduce the risk of overfitting, a leave-one-out sixfold cross-validation procedure was applied. In each iteration, trained TRFs were used to predict the left-out EEG response based on the corresponding stimulus. The predicted EEG response was then correlated with the actual EEG response to derive a prediction accuracy for each test trial and EEG channel in order to evaluate the forward model performance. To account for differences in the stimulus characteristic between phoneme and word regressor our correlation analysis only included time points 0 to 500 ms after the onset. In this way the correlation analysis between predicted and actual EEG response was based on valid time points only where a prediction could be made.

#### Regularization parameter tuning

A regularization (ridge) parameter was tuned to control for overfitting (Crosse et al., 2021), using values between 10^−6^ and 10^6^. For each subject, we fitted a forward model containing the phoneme and word onset regressors for the attended and ignored speech stream to determine the optimal ridge parameter by minimizing any bias due to selective auditory attention effects. The ridge parameter value corresponding to the TRF that produced the lowest mean square error, averaged across test trials and channels, was selected as the optimal regularization parameter for this subject and was used in each of the next analysis steps.

#### Channel selection

To minimize any bias of selective auditory attention effect on channel selection, we fitted a forward model containing the phoneme and word onset regressors for the attended and ignored speech stream. Additionally, by including phoneme as well as word onset regressors within the same forward model, this procedure ensures that the electrode selection captures auditory processes related to phoneme and word onset processing reasonably well. The prediction accuracy values were averaged across trials and subjects to derive a grand average prediction accuracy representation across channels. Based on the grand average prediction accuracy topography we followed a conservative data-driven approach and focused our analysis on six bilateral pairs of frontotemporal well-predicted channels. The selected channels resembled a plausible region of interest in accordance with bilateral generators in the auditory cortices, in line with recent research on neural speech tracking (Broderick et al., 2022; Di Liberto et al., 2015, 2018; Verschueren et al., 2022). These channels were used to average the TRF and prediction accuracies in each participant.

#### Model selection

In order to disentangle selective attention effects on the phoneme and word processing level we fitted three different models separately for the attended and ignored speech stream. The first model was based on the word onsets and served as a basis model to understand how well word onsets are tracked by the brain. The second model was based on the phoneme onsets. Here its important to note that the first phoneme of a word also corresponds to the word onset, but we do not provide this information to the model but rather deliver the phoneme onset information independent of the specific phoneme position in a word. This model was chosen to understand if phoneme onset information in general improves the prediction accuracy beyond the basic word onset model. In the last model, we included word and phoneme onset regressors to assess their independent contributions and determine whether additional word onset information provides an added value beyond phoneme onset processing and further improves the overall prediction accuracy. Based on this model we investigated the neural response to phoneme onsets and word onsets by examining the TRFs separately for the attended and ignored speech stream. To control for basic acoustic properties of speech, we conducted all forward model analyses both with and without including the speech envelope as an additional regressor.

### Statistical Analysis

All statistical analysis steps were performed in MATLAB (R2020a; MathWorks, 2020). We calculated the significance level of the prediction accuracy and TRFs by examining the forward model performance to shuffled versions of the word and phoneme onset regressor. For each speech stream, we created 100 unique permutations in which the word onsets and corresponding phoneme onsets were randomly rearranged within the permuted regressor. Importantly, the interword and interphoneme duration information was completely preserved. This permutation method ensures that the significance level calculation is based on speech-related plausible versions of the onset regressors. All of the 100 unique permutations had identical word and phoneme rate statistics compared to the original word and phoneme regressors. For each of the 100 permutations we calculated the forward model performance in all participants and averaged the TRFs and prediction accuracy across participants to create a chance level distribution for each model and attention condition. To verify that forward model prediction accuracy exceeded the chance level distribution, we performed two-sided Wilcoxon rank sum tests using a significance level of *α* = 0.05.

To investigate selective auditory attention effects, represented in the TRFs to the attended and ignored speech stream, we determine at which time points the attended and ignored TRF significantly differs from the chance level distribution by using a cluster-based permutation test as recommended by Maris and Oostenveld (2007). We used a running two-sided independent *t* test (cluster-forming threshold of uncorrected *α* = 0.001) on the difference TRF (attend–ignore) and compared it to the difference chance level TRF (attend–ignore) across all time lags (0–500 ms). For each cluster, the sum of all *t* values in the cluster is compared to a null distribution based on the largest cluster sum in 100,000 permutations of the data. To determine significant clusters, we used a corrected significance level of *α* = 0.001. To identify selective auditory attention effects represented in the prediction accuracy we performed two-sided Wilcoxon signed-rank tests between the different forward models and separately for the attend and ignore condition using a significance level of *α* = 0.05. Benjamini-Yekutieli correction (Benjamini & Yekutieli, 2001) was applied to correct for multiple comparisons and the adjusted *p* value is reported.

## RESULTS

### TRF

**Figure 3.**
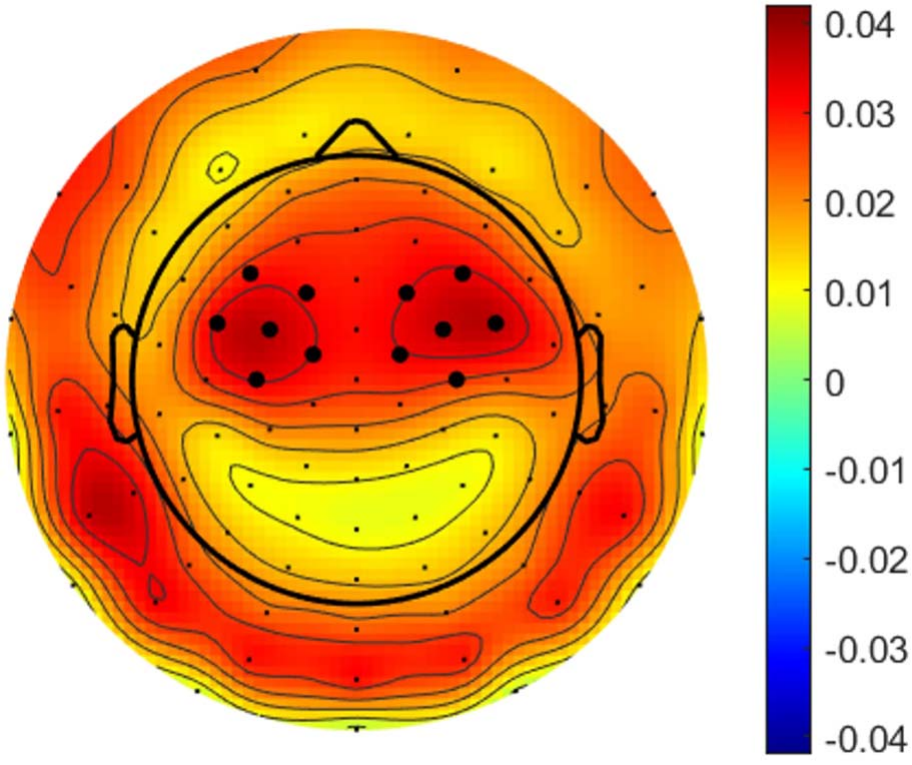
Grand average prediction accuracy topography. Six bilateral pairs of frontotemporal well-predicted channels were used to average the prediction accuracy and temporal respons function in each subject.

**Figure 4.**
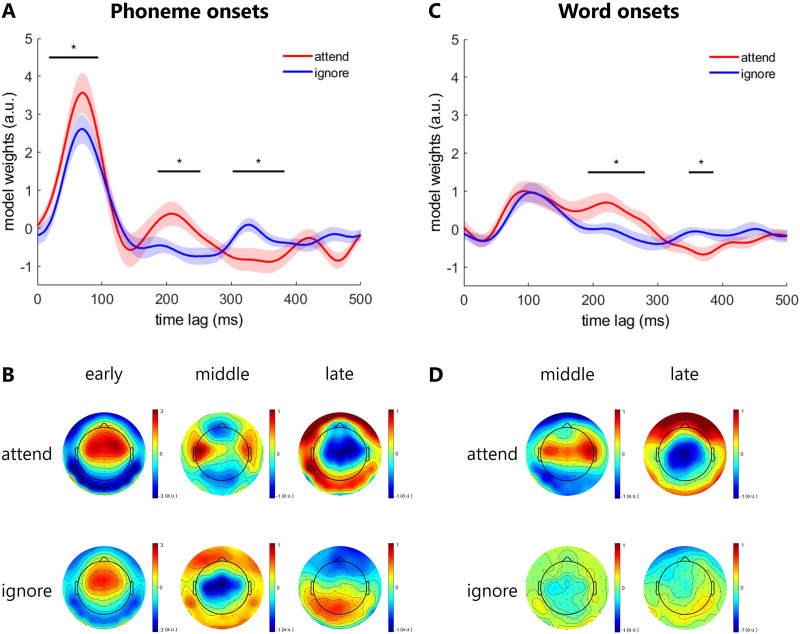
Grand average temporal response function (TRF) waveforms and topographic maps to phoneme and word onsets. (A) Grand average TRF waveform for attend and ignore phoneme onsets. Black lines above the waveform indicate the time lags where the TRF for attended speech is statistically different from the TRF for ignored speech across subjects (*p* < 0.001, running two-sided *t* test, cluster-based multiple comparison corrected). (B) Grand average topographic maps of the phoneme onsets TRF for attended and ignored speech at early (18–94 ms), middle (186–252 ms), and late (302–382 ms) attention effect. (C) Grand average TRF waveform for attend and ignore word onsets. Black lines above the waveform indicate the time lags where the TRF for attended speech is statistically different from the TRF for ignored speech across subjects (*p* < 0.001, running two-sided *t* test, cluster-based multiple comparison corrected). (D) Grand average topographic maps of the word onsets TRF for attended and ignored speech at middle (192–280 ms) and late (348–386 ms) attention effect.

To understand how selective auditory attention influences the lexical speech segmentation process in a two competing speaker scenario, we investigated the neural response to phoneme and word onsets by examining the TRFs separately for the attended and ignored speech stream. The TRFs were averaged across six bilateral pairs of frontotemporal channels that showed the highest prediction accuracies (see Figure 3). The distribution of the EEG grand average prediction accuracies corresponds well with previous research (Di Liberto et al., 2015). To control for basic acoustic properties of speech, we conducted the forward model analysis both with and without including the speech envelope as an additional regressor. As both analyses resulted in similar TRF time courses and attention effects (see Supplementary Figure S6 in the Supporting Information, available at https://doi.org/10.1162/nol.a.14), we report in Figure 4 the results where the speech envelope was included in the forward model analysis.

Figure 4A illustrates the TRF to phoneme onsets in the attend (red) and ignore (blue) condition. The TRF model weights are plotted against the time lag (in ms), ranging from 0 to 500 ms relative to phoneme onset. Both conditions show an initial positive peak up to a time lag of 100 ms. A running two-sided *t* test identified a significant difference between the conditions, apparent from 18 ms to 94 ms indicating that the response in the attend condition is significantly greater than in the ignore condition within this early time window. The corresponding topographic maps (Figure 4B, left panel) indicate higher model weights over frontocentral electrode sites in the attend compared to the ignore condition. Another notable period where the attend condition shows a significantly higher response compared to the ignore condition based on a running *t* test is evident at a middle time window from 186 ms to 252 ms. Here, the corresponding topographic maps (Figure 4B, middle panel) indicate a more focal bilateral pattern toward temporal electrode sites with modest left lateralization in the attend condition and widespread negative model weights over frontocentral sites for the ignore condition. Finally, the running two-sided *t* test identified a significant difference between the conditions, apparent from 302 ms to 382 ms, indicating that the response in the attend condition is significantly more negative than in the ignore condition within this late time window. The corresponding topographic maps (Figure 4B, right panel) indicate widespread negative model weights over frontocentral electrode sites in the attend condition and focal positive model weights over occipital sites for the ignore condition. Beyond a time lag of 400 ms, the TRF weights for both conditions fluctuate around zero with no clear peaks or significant differences between the attend and ignore conditions. The differences observed between the attend and ignore conditions suggest that selective auditory attention modulates the neural response to phoneme onsets, particularly within an early and late time window.

Figure 4C illustrates the TRF to word onsets in the attend (red) and ignore (blue) condition. Both conditions show an initial peak up to a time lag of 150 ms, with similar amplitudes for the attend and ignore condition. Based on a running two-sided *t* test there is no significant difference in the model weights between the conditions in this early time window. After 150 ms, a divergence between the two conditions can be observed. The attend condition shows a positive peak, which is noticeably higher than the response in the ignore condition. This difference is statistically significant in a middle time window from 192 ms to 280 ms based on a running two-sided *t* test. The corresponding topographic maps (Figure 4D, left panel) indicate a more focal bilateral pattern toward temporal electrode sites with modest right lateralization in the attend condition and a focal bilateral pattern of slightly negative model weights for the ignore condition. Finally, the running two-sided *t* test identified a significant difference between the conditions, apparent from 348 ms to 386 ms, indicating that the response in the attend condition is significantly more negative than in the ignore condition within this late time window. The corresponding topographic maps (Figure 4D, right panel) indicate widespread negative model weights over centroparietal electrode sites in the attend condition and focal, slightly positive model weights over occipital sites for the ignore condition. Beyond 400 ms, the TRF model weights for both conditions fluctuate and eventually converge around zero with no significant differences between them. The significant difference observed in the middle and late time window suggests that selective auditory attention modulates the neural response to the word onsets, with heightened neural activity when the speech stream is attended to compared to when it is ignored.

### Prediction Accuracy

**Figure 5.**
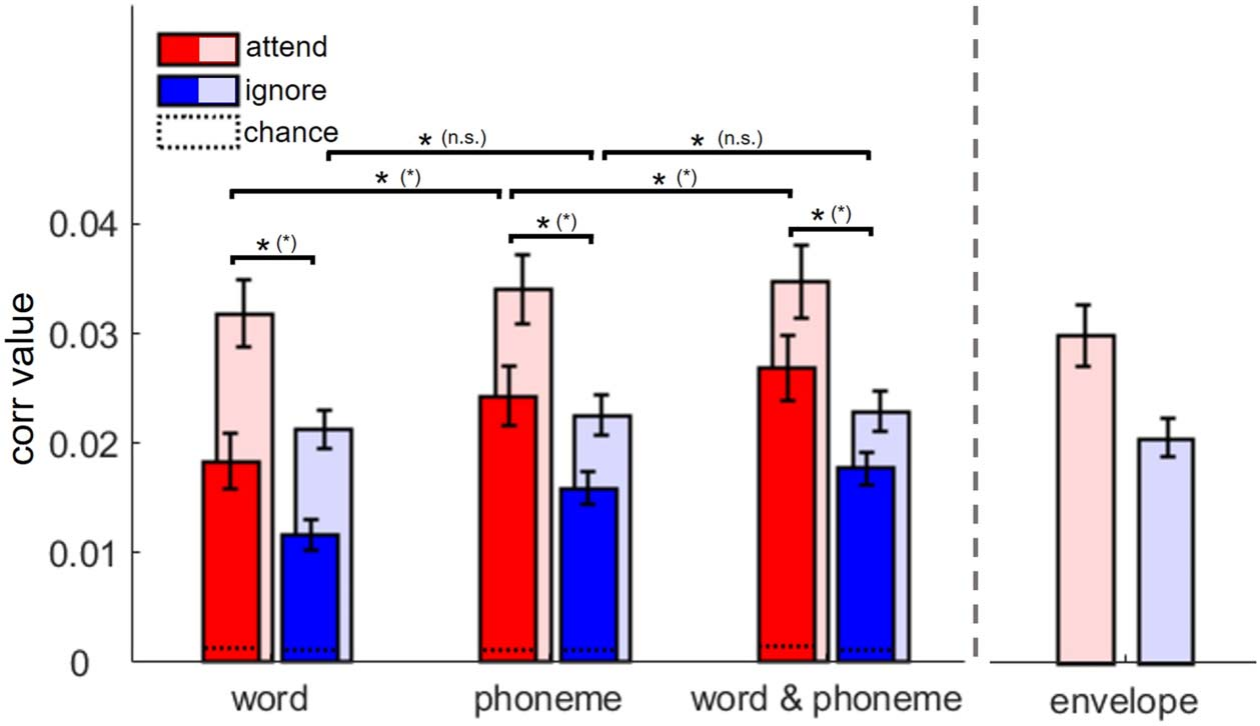
A cross-validation procedure was used to predict EEG responses to the attended and ignored speech using only word, only phoneme, and word and phoneme predictor variables. Solid red and blue bars indicate model prediction accuracy without including the speech envelope, while the lighter red and blue bars show the corresponding accuracy when the speech envelope is included as an additional regressor. Black lines and asterisks indicate statistically significant differences between model prediction accuracies (*p* < 0.05, two-sided Wilcoxon signed rank test, false discovery rate corrected, error bars represent standard error of the mean). In brackets, the statistical outcome when including the speech envelope as an additional regressor is shown. On the far right, model prediction accuracy using only the speech envelope regressor is shown.

Figure 5 presents the prediction accuracy represented in the correlation value in the three different forward models, including word, phoneme, and word and phoneme onset regressors, separately for the attend (red) and ignore (blue) condition. Solid red and blue bars in the front represent the model prediction accuracies without including the speech envelope, and lighter red and blue bars in the back represent the corresponding accuracies when the speech envelope is included as an additional regressor to control for the basic acoustic properties of the speech. The rightmost pair of bars represents model prediction accuracy using only the speech envelope regressor. These envelope-only results are shown for illustrative purposes to provide a baseline but were not included in further statistical analyses, as our focus was on the word, phoneme, and combined word and phoneme models.

In all forward models, with and without including the speech envelope, prediction accuracy significantly exceeded their respective chance level. Remarkably, the chance level prediction accuracy stays on a low stable level independent of the specific forward model and number of regressors included in the model. On a descriptive level, including the speech envelope as an additional regressor in the forward model results in higher prediction accuracies compared to the forward model without including the speech envelope. There is a significant difference in prediction accuracies between the attend and ignore conditions represented in all forward models. With and without including the speech envelope, correlation values were higher for the attend compared to the ignore condition (without speech envelope: word: Z = 2.65, *p* = 0.024; phoneme: Z = 2.91, *p* = 0.013; word and phoneme: Z = 3.06, *p* = 0.01; with speech envelope: word: Z = 3.21, *p* = 0.006; phoneme: Z = 3.39, *p* = 0.004; word and phoneme: Z = 3.50, *p* = 0.004). Taken together, in all forward models, with and without including the speech envelope, a clear effect of selective auditory attention on prediction accuracy is evident.

When comparing the prediction accuracies across the different forward models, without including the speech envelope in the analysis, a clear significant increase from word to phoneme model (attend: Z = 3.62, *p* = 0.003; ignore: Z = 2.50, *p* = 0.032) as well as from phoneme to word and phoneme model (attend: Z = 3.73, *p* = 0.003; ignore: Z = 3.17, *p* = 0.009) is evident. This pattern is represented in the attend and in the ignore condition. In contrast, when comparing the prediction accuracies across the different forward models, with inclusion of the speech envelope, a clear significant increase from word model to phoneme model (Z = 3.73, *p* = 0.003) as well as from phoneme model to word and phoneme model (Z = 2.87, *p* = 0.015) is evident in the attend condition, but not in the ignore condition (word to phoneme: Z = 2.13, *p* = 0.1; phoneme to word and phoneme: Z = 1.94, *p* = 0.14).

## DISCUSSION

### Summary and Main Finding

In this study we investigated EEG responses to continuous speech streams to understand how selective auditory attention influences the lexical speech segmentation process in a two competing speaker scenario. We examined the neural responses to phoneme and word onsets by analyzing TRFs separately for the attended and ignored speech stream. Selective auditory attention enhanced the neural response to phoneme onsets, with significant differences occurring in early (18–94 ms), middle (186–252 ms), and late (302–382 ms) time windows, particularly over frontocentral (early and late attention effect) and bilateral temporal electrode sites (middle attention effect). For word onsets, selective auditory attention enhanced the neural response only in middle (192–280 ms) and late (348–386 ms) time windows, showing more focal activity over bilateral temporal (middle time window) and centroparietal (late time window) electrode sites. Model prediction accuracy, without including the speech envelope, was higher for the attended speech stream across all models, but significant improvements from word to phoneme model, and from phoneme to word and phoneme model was found for the attended and ignored speech stream. In contrast, with including the speech envelope, a significant improvement from word to phoneme, and from phoneme to word and phoneme model was found for the attended but not for the ignored speech stream.

### TRF

We have shown that, in a two competing speaker scenario, paying attention to one speaker influences ongoing cortical activity related to phoneme and word onsets, reflecting selective auditory attention. The observed TRF time courses are similar to recent research on continuous speech processing with a single speech stream (Agmon et al., 2023; Brodbeck et al., 2018; Broderick et al., 2018; Di Liberto et al., 2015; Donhauser & Baillet, 2020; Gillis et al., 2021). Especially in the attend condition we found for phoneme and word onsets robust TRF responses with peaks at time lags corresponding well to the auditory P1, P2, and N2 components from recent speech tracking literature (Horton et al., 2013; Jaeger et al., 2020; Kong et al., 2014; Mirkovic et al., 2019; Petersen et al., 2017). Inspection of the TRF in the ignore condition suggested that selective attention had a major impact on phoneme and word onset processing reflected in a reduced P1, P2, and N2 amplitude to phoneme onsets and a reduced P2 and N2 amplitude to word onsets compared to the attend condition. This general modulatory effect is in line with top-down attention gain control ideas and indicates a stronger cortical phase-locking to the attended compared to the ignored speech stream (Ding & Simon, 2012; Fiedler et al., 2019; Horton et al., 2013; Jaeger et al., 2020; Kerlin et al., 2010; Kong et al., 2014; Mesgarani & Chang, 2012; Zion Golumbic et al., 2013).

Whereas the general TRF time course seems to be similar between phoneme and word onsets, the found selective attention effects differ in latency and topographic characteristics. For phoneme onsets we found an early (P1, 18–94 ms), middle (P2, 186–252 ms), and late (N2, 302–382 ms) attention effect component. While the P1 component in the ignore condition is slightly reduced in amplitude compared to the attend condition, the P2 and N2 components seem to be strongly suppressed. For word onsets there was only a middle (P2, 192–280 ms) and a late (N2, 348–386 ms) attention effect, and these effects seem to be slightly later compared to the phoneme onsets. Our results suggest that TRFs to phoneme and word onsets may reflect different stages of speech processing and that selective auditory attention may affect each of these stages differently, in line with recent research (Brodbeck et al., 2018; Broderick et al., 2018; Mesik et al., 2021).

The selective auditory attention studies by Brodbeck et al. (2018), Broderick et al. (2018), and Mesik et al. (2021) are challenging to compare due to differences in predictor variables and the complexity of linear regression models, each using different combinations of acoustic, lexical, and linguistic features. However, collectively, these studies provide insight into how selective auditory attention may influence lexical speech segmentation. Mesik et al. (2021) examined attention effects on word features such as word onset, audibility, semantic dissimilarity, and surprisal, finding stronger TRF amplitudes for attended speech around 100 ms to word onsets, with higher order effects (semantic dissimilarity, surprisal) emerging later at 200–600 ms. Brodbeck et al. (2018) included a wide range of features and found that only word onset and phoneme cohort entropy significantly contributed to model predictions, with TRF peaks in attended speech at approximately 118 ms (word onset) and 140 ms (phoneme cohort entropy). Broderick et al. (2018) focused on word semantic dissimilarity, observing a larger TRF negativity for attended speech at 380–600 ms compared to ignored speech. Together, these findings suggest that speech perception operates within a hierarchical auditory processing system, with selective attention effects occurring at progressively later stages for increasingly complex speech attributes. Our data support this view by showing a slightly earlier P2 and N2 attention effect in the TRF to phoneme onsets compared to word onsets. Similar to Brodbeck et al. (2018), the P2 to word and phoneme onsets were strongly suppressed in the ignore condition indicating that lexical processing is reduced for the ignored speech stream. Additionally, the N2 attention effect to word onsets compares well in latency and topographic characteristic to Broderick et al. (2018), indicating a suppressed higher order word processing.

On a descriptive level, the topography at around 200–300 ms (P2, middle attention effect) in the attend condition differed between phoneme and word onsets. For phonemes we found a bilateral focal pattern with modest left lateralization while word onsets elicited a bilateral focal pattern with modest right lateralization. These findings are in line with the asymmetric sampling in time hypothesis, which proposes that speech perception is mediated bilaterally, with left nonprimary auditory areas preferentially extracting information from short (∼20–50 ms) temporal integration windows while the nonprimary right auditory areas preferentially extract information from long (∼200–300 ms) integration windows (Poeppel, 2003). Short temporal integration windows are relevant for encoding rapidly changing spectral information in speech (e.g., rapid formant transitions in the context of place-of-articulation differences) important for phoneme processing, while long temporal integration windows are required to encode slowly changing spectral information in speech as represented in words and syllables. Our approach based on time-aligned phoneme and word onsets may capture this functional segregation and could potentially be used to explore changes in hemispheric asymmetries in speech processing due to aging, hearing loss, effects of plasticity, and training (Firszt et al., 2006).

In our study, we found a weak early attention effect (P1) and a strong middle (P2) and late (N2) attention effect for phoneme onsets, while word onsets showed only a strong middle (P2) and late (N2) attention effect. Apart from the unexpected middle and late effect for phoneme onsets, these results align with the hierarchical speech processing model (Hickok, 2022; Hickok & Poeppel, 2007), which predicts early attention effects for phonemes and later effects for words. Further, the conceptual model of early speech processing, proposed by Bronkhorst (2015), may help to explain our differences between the early and later attention effects. The model states that speech processing is largely feedforward but influenced by an attention-controlled feedback loop that causes selective enhancement of the relevant speech input. Attention can act on multiple levels: two early (fast selection) levels and a late (slow selection) level. Fast selection can be driven by bottom-up and/or top-down processes but is in both cases based on basic speech features, while slow selection operates at higher order levels, processing more complex information like lexical, semantic, and syntactic features. The model also proposes that selective enhancement is largest at late or higher order stages of speech processing. Our data align with this model. The weaker early attention effect on phoneme onsets reflects early top-down selection based on basic or more primitive acoustic phoneme features. The stronger middle and late selective attention effects for both phoneme and word onsets may reflect higher level speech processing, like lexical segmentation, which are more strongly suppressed for the ignored speech stream. This is also consistent with empirical results by Brodbeck et al. (2018) showing that selective attention suppresses the lexical processing of the ignored speech and with Brodbeck et al. (2018), who found reduced semantic processing for the ignored stream. Additionally, our findings support the “cocktail party effect,” where people struggle to report the content of ignored speech (Cherry, 1953) but still recognize relevant information, such as hearing their own name (Holtze et al., 2021). This suggests that weaker attention effects at early processing levels allow for some semantic processing of ignored speech, triggering this recognition.

One possible explanation for the unexpected middle and late attention effects for the phoneme onsets may be that speech processing is based on a complex network analyzing phonological, lexical, and semantic information in parallel (Giraud & Poeppel, 2012; Pulvermüller & Fadiga, 2010). Recent research revealed that lexical segmentation effects emerge at overlapping time windows for phonemes and words (Broderick et al., 2018; Mesik et al., 2021). This suggests that speech processing may be not as temporally segregated as traditionally assumed but instead involve parallel mechanisms. Finally, a recent magnetoencephalography study by Brodbeck et al. (2022) based on a single speech stream, found that robust speech processing in the auditory system is realized by multiple context models based on phoneme-, word-, and sentence-level information that process the speech input in parallel and by cutting across hierarchical levels of representation. Their results further suggest that the different context models are maintained by at least partially separable neural processes as they originated from different source configurations in the right and left hemisphere of the brain. We do not know yet how selective attention in a two competing speaker paradigm would affect the processing in each of these context models, though it may be the case that we captured with our linear regression model based on phoneme and word onsets separate context models and therefore found a strong middle and late selective attention effect for word as well as for phoneme onsets. Taken together, our findings align with a hierarchical model of speech processing (Hickok & Poeppel, 2007), where phoneme processing typically occurs earlier (50–100 ms) and word-level processing engages later (200–400 ms). Observing late phoneme attention effects suggests that phoneme-level processing may not be purely feedforward but rather influenced by contextual or lexical feedback loops (Brodbeck et al., 2022). This would suggest that phoneme processing is dynamically integrated with word-level and higher order linguistic features to enable robust speech processing. Future research should investigate whether late phoneme attention effects are influenced by lexical context or top-down semantic feedback, possibly using nonlinear modeling approaches to capture more complex neural interactions.

### Prediction Accuracy

In line with models of hierarchical speech processing and previous studies (Brodbeck et al., 2018; Broderick et al., 2018; Mesik et al., 2021) we expected lexical segmentation to yield a stronger increase in prediction accuracy in the attended compared to the ignored speech stream. Indeed, with and without including the speech envelope, we found for the attended compared to the ignored speech stream higher model prediction accuracies across all models. Without including the speech envelope, significant improvements from word to phoneme model, and from phoneme to word and phoneme model were found for the attended and ignored speech stream. In contrast, with including the speech envelope, a significant improvement from word to phoneme model, and from phoneme to word and phoneme model was found for the attended but not for the ignored speech stream. The general selective attention effect replicates in strength and direction typical results of recent research (Brodbeck et al., 2018; Broderick et al., 2018; Mesik et al., 2021). Together with our TRF results this indicates a stronger cortical phase locking to the attended compared to the ignored speech stream. Its important to note that due to our correlation analysis based on valid time points in the range of time lag after the respective onsets the increase in prediction accuracy cannot be explained by differences in regressor characteristics (e.g., the number of onsets or differences in interonset duration). This is also supported by our permutation analysis that delivered similar low chance levels across all forward models. By preserving interword and interphoneme durations in the shuffled regressors, our permutation procedure ensured that the prediction accuracy was not driven by spurious temporal correlations.

The higher prediction accuracy for phoneme onsets over word onsets underscores the significance of phoneme onset information within words for speech tracking. Since our regressor included identical information at both word and first phoneme onsets, this accuracy boost likely stems from the additional phoneme onsets within words, indicating a relevant neural response to these onsets. Moreover, phoneme and word onset information combined explained more neural response than either alone, suggesting distinct brain responses to each, reflected by differences in TRF amplitude and latency. The limited accuracy gain from combining phoneme and word onsets implies that their information largely overlaps, rather than being additive. Phoneme onsets inherently contained word onset information, as the first phoneme of each word corresponded to the word onset. However, word onsets introduce additional linguistic segmentation cues, but since phoneme onset information was already strongly represented, the added value of explicitly including word onsets was limited.

Based on models of hierarchical speech processing we expected effects of lexical speech segmentation to be reflected as a stronger increase in prediction accuracy in the attended compared to the ignored speech stream. Especially, when comparing the phoneme onset and word and phoneme onset model to the ignored speech stream, we expected no further increase in prediction accuracy, indicating suppressed higher level word processing. Interestingly, our analysis with and without including the speech envelope as an additional regressor revealed mixed results. Without including the speech envelope in the forward model, significant improvements in prediction accuracy were evident for the attended and ignored speech stream, which is in contrast to our hypothesis. With inclusion of the speech envelope, significant improvements were only evident for the attended speech stream but not for the ignored speech stream, which is in favor of our hypothesis. Two aspects could potentially explain the conflicting finding.

Without including the speech envelope, a possible explanation for the increase in prediction accuracy may be that higher level processing for the ignored speech stream is reflected in a phase-locked active suppression mechanism. Especially at the middle time interval corresponding to the P2 component we found for phoneme as well as for word onsets negative TRF weights at electrode sites plausible for auditory processing. This inverse pattern may indicate a phase-locked mechanism actively suppressing the processing of the ignored speech stream. Our finding is consistent with recent research that indicates separate active enhancement and suppression mechanisms for attended and ignored speech as a general principle of selective auditory attention (Fiedler et al., 2019; Horton et al., 2013; Kong et al., 2014). Especially in high-demanding listening situations, such as the competing speaker paradigm, ignoring the irrelevant aspects of an auditory scene is at least as important as attending to the relevant aspects in order to increase the signal-to-noise ratio between the attended and ignored speech information in the auditory system sufficiently (Bidet-Caulet et al., 2007; Jaeger et al., 2018). Indeed, Fiedler et al. (2019) found under adverse listening conditions an enhanced response to the ignored talker in a later time range between 200 ms and 300 ms corresponding to our P2 attention effect. Their analysis revealed enhanced neural selective processing of the ignored talker in nonauditory regions, which are part of the frontoparietal attention network. Neural signatures for active suppression of irrelevant sounds during late (200 ms) auditory evoked potentials have been described before (Chait et al., 2010; Melara et al., 2002), and it can be assumed that such an active suppression mechanism puts additional demands on the cognitive system and is only deployed if needed (Chait et al., 2010). In our study, we derived separate forward models for the attended and the ignored speech stream in order to disentangle selective auditory attention effects. An active suppression mechanism for the ignored speech stream would still result in an increase in prediction accuracy if the mechanism is phase locked to the ignored speech stimulus. However, this increase in prediction accuracy should be interpreted with caution. Given our bilateral frontotemporal channel selection, it does not necessarily indicate an improved encoding of the ignored speech stream in the auditory cortex alone but may capture enhanced neural activity in nonauditory regions such as the frontoparietal attention network. Furthermore, our TRF analysis based on time lags from 0 ms to 500 ms does not allow for final conclusions about which time intervals or hierarchical levels of speech processing contribute most to this effect. Therefore, further research is needed to confirm this effect and to determine how cognitive load may influence it by incorporating behavioral measures such as speech intelligibility.

Including the speech envelope as an additional regressor in the forward model analysis may cover these effects. The speech envelope tracks the slow amplitude fluctuations of the speech signal and is often used to control for basic acoustic properties of speech, in order to disentangle low-level acoustic processing from higher level linguistic processing (Brodbeck et al., 2018; Daube et al., 2019; Gillis et al., 2021). Due to its characteristics, the speech envelope inherently contains additional information about word and phoneme onsets, as sudden amplitude changes in the envelope time course reflect transitions between words, syllables, or phonemes (e.g., consonants vs. vowels). It is likely that our prediction accuracies were influenced by this additional onset information present in the speech envelope, which may explain the relatively smaller increase in accuracy from the word to the phoneme model and to the combined word and phoneme model. This pattern was observed for both the attended and ignored speech streams. The added value of explicitly incorporating word and/or phoneme onset information may have been lower due to its overlap with the onset information already embedded in the speech envelope. Taken together, when analyzing neural tracking of higher level speech processes, controlling for low-level acoustic properties by including the speech envelope can help avoid spurious significant findings related to higher level linguistic features. However, selecting an optimal control regressor that purely reflects acoustic properties of speech is challenging, if not impossible, as low-level acoustic and higher level linguistic features are often correlated. Future studies could explore this issue further, as the choice of the optimal control regressor likely depends on the specific research question and the speech features used in the analysis.

### Conclusion

This EEG study explored how selective auditory attention affects neural responses to phoneme and word onsets in continuous speech. The results show that attention enhances the neural tracking of the attended speech stream while suppressing responses to the ignored speech. The distinct early, middle, and late effects observed for phoneme onsets, alongside the middle and late effects for word onsets, support the coexistence of hierarchical and parallel processing models of speech processing. Selective auditory attention also improved lexical segmentation, as reflected in increased prediction accuracy for the attended speech stream. By combining phoneme and word onset models, neural tracking was further improved. By providing neural evidence for how attention modulates speech processing at different linguistic levels, this study contributes to a deeper understanding of selective auditory attention and its influence on hierarchical speech segmentation mechanisms. Beyond theoretical contributions, these findings have potential clinical applications for individuals with auditory processing disorders, hearing impairments, or difficulties in multispeaker environments. Understanding how attention modulates phoneme and word segmentation may inform the development of attention-based auditory training programs aimed at improving speech perception in challenging listening conditions. Overall, this study highlights the crucial role of selective attention in modulating speech processing at different hierarchical levels, reinforcing its importance in speech comprehension within complex auditory environments.

## ACKNOWLEDGMENTS

We would like to thank Till Eric Wagner for his assistance in speech annotation, Thorge Haupt and Sebastian Puschmann for valuable discussions on data analysis.

## FUNDING INFORMATION

Elana Zion Golumbic, Israel Science Foundation (https://dx.doi.org/10.13039/501100003977), Award ID: 2339/20. Martin G. Bleichner, Deutsche Forschungsgemeinschaft (https://dx.doi.org/10.13039/501100001659), Award ID: 490839860. Martin G. Bleichner, Deutsche Forschungsgemeinschaft (https://dx.doi.org/10.13039/501100001659), Award ID: 411333557.

## AUTHOR CONTRIBUTIONS

**Manuela Jaeger**: Conceptualization; Methodology; Writing – original draft; Writing – review & editing. **Elana Zion Golumbic**: Conceptualization; Funding acquisition; Methodology; Writing – original draft; Writing – review & editing. **Martin G. Bleichner**: Conceptualization; Funding acquisition; Methodology; Writing – original draft; Writing – review & editing.

## AI USAGE

During the preparation of this work, the author(s) used ChatGPT 4o and the free version of ChatGPT (mid 2024) in order to improve language and readability of selected sentences. After using this tool/service, the author(s) reviewed and edited the content as needed and take(s) full responsibility for the content of the publication.

## DATA AND CODE AVAILABILITY STATEMENT

Preprocessed data and analysis scripts are available in a Zenodo repository (https://doi.org/10.5281/zenodo.15518572).

## Supplementary Material


