# Supplementary material for: Attention Modulation to Linguistic Speech Units
Source: Neurobiol Lang (Camb). 2025 Sep 5;6:nol.a.14. doi: 10.1162/nol.a.14 (PMC12435783; doi:10.1162/nol.a.14)
Supplement: Supplementary file 1 [file nol-6-1-14-s001.pdf]

Supplementary

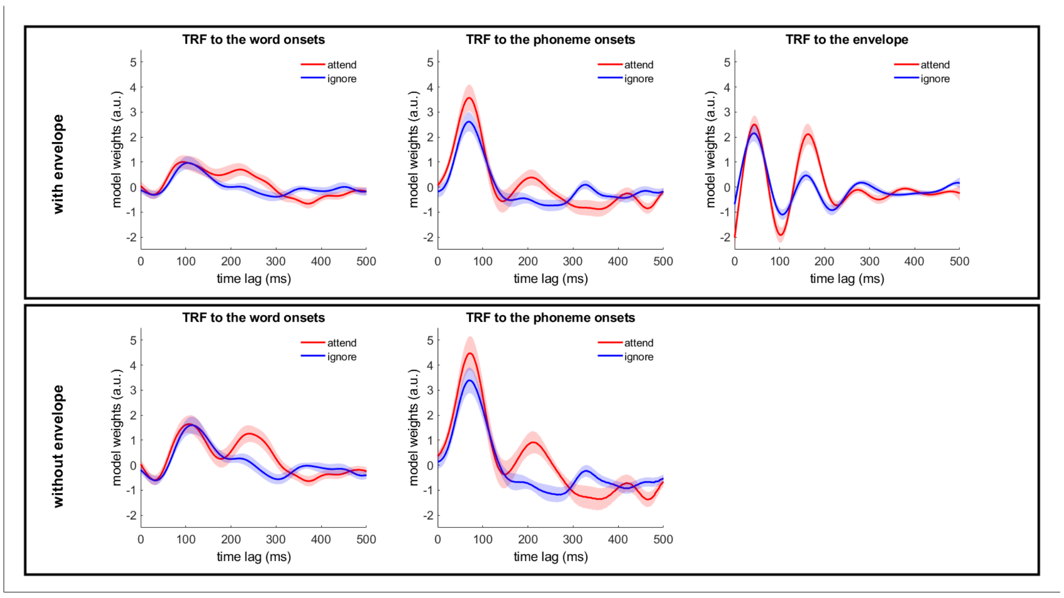

Figure 6: Grand average TRF waveforms for word onsets, phoneme onsets, and the speech envelope, shown separately for the attended (red) and ignored (blue) speech stream. The upper panel shows TRFs from the combined word & phoneme model that includes the speech envelope as an additional regressor to control for basic acoustic properties of speech. The lower panel shows corresponding TRFs from the same model without the inclusion of the speech envelope.
